# Death from the Administration of Chloroform—Autopsy

**Published:** 1854-01

**Authors:** 


### ARTICLE XIII.

*Death from the Administration of Chloroform——Autopsy.* Under the care of Mr. PAGET, of St. Bartholomew's Hospital, London.

THE following are the particulars of the case of death from chloroform, to which we alluded last week. In reference to the administration of the agent, we may state, that at St. Bartholomew's it is, by appointment, always confided to the very able charge of Dr. Black, one of the assistant physicians.

On Thursday afternoon, Oct. 20, it was intended to apply the actual cautery to a sore of cancroid nature in the vagina of a patient named Ann Smith, aged 22 years. She was a stout, florid young woman, formerly of dissolute habits, but apparently, with the exception of the local ailment, in perfect health; she had been in the hospital several months, and, about a fortnight previously, had been put under the full and prolonged influence of chloroform for a like purpose, and without the occurrence of any untoward symptoms whatever. Her pulse was regular, of good power, and average frequency. It had been ordered that she should omit her dinner on the day of the operation, but, as discovered at the *post-mortem*, she had, unknown to the nurses, taken a quantity of food, though not a full meal.

The usual form of inhaler was employed,—a padded metal cup, fitting over the nose and mouth, and supplied with valves. A drachm, by measure, was first poured on the sponge, but, as the administration did not immediately commence, a considerable part of this was no doubt wasted; after a short inhalation, a second drachm was supplied, and subsequently the further quantity of half a drachm. The patient had gone through the usual stages of excitement, etc., and the last dose was scarcely used, as she sank off, almost immediately after its application, into a state of complete insensibility, unattended by any alarming symptoms. About five minutes had been occupied in the inhalation, and probably not more than a drachm and a half of the fluid really inhaled. The apparatus was now removed from the face, and the patient having been drawn into the proper position, Mr. Paget was about to commence the operation, when Dr. Black, who throughout had kept his finger on the pulse, noticed it to have become extremely feeble and fluttering. Almost immediately afterwards the patient's countenance was observed to be dusky, turgid, and congested, and the respiratory movements began to be performed at long intervals, and by slight catching efforts. No time was lost; cold water was at once dashed on the thighs, face, and breast, and, the failure in the respiration becoming shortly complete, Mr. Paget immediately began artificial insufflation of the lungs by alternately blowing into the nostrils, and compressing the chest. Just before commencing this process, Mr. Paget had ascertained, by drawing the tongue forward and examining the glottis with the finger, that the epiglottis was not pressed down. Artificial respiration through the natural passages having been very efficiently kept up for about ten minutes, the nose appeared to have got clogged, and Mr. Paget accordingly performed tracheotomy in order to permit of the more free introduction of air into the lungs. A brandy enema was administered, and, within ten minutes of the seizure, galvanism was also put in use, but without any good result, and after about three-quarters of an hour had been spent in persevering efforts to produce reanimation, they were laid aside as hopeless. It was noticed, that immediately after the first alarming symptoms, the pupils were of medium size, neither contracted nor dilated. All efforts at respiration ceased about two minutes after the first indications of failure; the pulse, however, as a very feeble flutter, was felt occasionally for at least two minutes later. Thus, then, the facts of the case were briefly these: A patient, in profound coma, from the administration of an anæsthetic, had, the exhibition of the drug having been remitted, been suddenly seized with symptoms of failing circulation, followed by the speedy suspension of respiratory efforts; the pulse had remained perceptible a little time after the cessation of the latter, and had been lost, together with every manifestation of vitality, within five minutes of the first indication of danger. Every effort to re-excite the play of the organs essential to life had been fruitless. In the turgidity and suffusion of the countenance there had been some indication of death by asphyxia. The preceding coma, and the fact that the failure of the pulse had been the first symptom exciting attention, each pointed to other conclusions. The brain, the heart, the lungs,—in which organ had death commenced? This seemed the important problem. Let us now see what evidence the *post-mortem* examination furnished for its solution.

*Autopsy performed by Mr. Paget twenty-two hours after Death.*—The countenance was still bloated and suffused; the *post-mortem* rigidity was moderate in degree, or rather less than usual; there was much congestion and lividity of the skin of the depending parts of the body; the corpse was very fat. The thorax was first examined, and nothing whatever abnormal could be detected in any of its viscera; the lungs were healthy and crepitant in every part; their posterior lobes were not more congested than is seen in almost every examination; the heart collapsed, but not contracted, and containing a small quantity of fluid blood in each cavity, was of normal size and proportions in every respect, and its muscular structure examined by the microscope showed no degeneration. Excepting that the exterior of the right kidney was puckered in places, as if from disease in early life, nothing worthy of note was observed in the abdominal viscera. The brain, its sinuses, ventricles, etc., were all carefully examined, and neither in texture nor quantity of blood was anything abnormal detected. The spine was not examined. In every part inspected—the heart, great vessels, lungs, vessels within the abdomen, those of the scalp, pia-mater, and brain—the blood was universally fluid, and without the slightest trace of coagulum, or even of inspissation. Collected in the quantity of an ounce or two, and allowed to stand in an open vessel, it did not coagulate, nor in any material degree change its dark purple color. Looking, then, at these facts, and comparing them with the symptoms manifest during life, we seem compelled to seek, in a humoral pathology, the cause of the patient's death. There was no visceral disease, functional or otherwise. In all probability the blood, poisoned past recovery by the vapor it had received, had died, and ceasing then to afford to any of the organs their natural stimulus, the whole of the vital functions had ceased almost coincidently.

This case, in common with most of those which occur in our Metropolitan hospitals, has one most especially melancholy aspect—a matter, as far as our knowledge goes, of pure accident—it teaches us nothing for the future. We need not recapitulate to convince the attentive reader that the patient was one to whom chloroform might, with the least of apprehension, be administered; she was in good health; she had taken it safely before. Then, again, in the manner of exhibition, every precaution was adopted, and, in the after-treatment, all that science could suggest was perseveringly tried. In our large hospitals the requisite skill, assistance, and appliances are always at hand; and there, if anywhere, an immunity from fatal results might be expected. It is mournful, indeed, to consider that from cases such as the last three or four, which it has been our lot to record, the practical surgeon gains no knowledge calculated to authorize the hope, that in future, the like tragedies will be of less frequent occurrence.—*London Medical Times and Gazette.*

